# Biomarkers of Acute Stroke Etiology (BASE) Study Methodology

**DOI:** 10.1007/s12975-017-0537-3

**Published:** 2017-05-05

**Authors:** Edward C. Jauch, Andrew D. Barreto, Joseph P. Broderick, Doug M. Char, Brett L. Cucchiara, Thomas G. Devlin, Alison J. Haddock, William J. Hicks, Brian C. Hiestand, Glen C. Jickling, Jeff June, David S. Liebeskind, Ted J. Lowenkopf, Joseph B. Miller, John O’Neill, Tim L. Schoonover, Frank R. Sharp, W. Frank Peacock

**Affiliations:** 1 Medical University of South Carolina, Charleston, SC USA; 2 University of Texas, Houston, TX USA; 3 University of Cincinnati, Cincinnati, OH USA; 4 Washington University, St. Louis, MO USA; 5 University of Pennsylvania, Pittsburgh, PA USA; 6 Erlanger Health System, Chattanooga, TN USA; 7 Baylor College of Medicine, 3302 S. Macgregor Way, Houston, TX 77021 USA; 8 OhioHealth Riverside Methodist Hospital, Columbus, OH USA; 9 Wake Forest, School of Medicine, Winston-Salem, NC USA; 10 University of California, Davis, Sacramento, CA USA; 11 Ischemia Care, LLC, Cincinnati, OH USA; 12 University of California, Los Angeles, CA USA; 13 Providence Brain and Spine Institute, Portland, OR USA; 14 Henry Ford Hospital, Detroit, MI USA; 15 Allegheny Medical Center, Pittsburgh, PA USA; 16 Dayton Center for Neurological Disorders, Centerville, OH USA

**Keywords:** Acute Stroke, RNA expression, Biomarkers

## Abstract

Acute ischemic stroke affects over 800,000 US adults annually, with hundreds of thousands more experiencing a transient ischemic attack. Emergent evaluation, prompt acute treatment, and identification of stroke or TIA (transient ischemic attack) etiology for specific secondary prevention are critical for decreasing further morbidity and mortality of cerebrovascular disease. The Biomarkers of Acute Stroke Etiology (BASE) study is a multicenter observational study to identify serum markers defining the etiology of acute ischemic stroke. Observational trial of patients presenting to the hospital within 24 h of stroke onset. Blood samples are collected at arrival, 24, and 48 h later, and RNA gene expression is utilized to identify stroke etiology marker candidates. The BASE study began January 2014. At the time of writing, there are 22 recruiting sites. Enrollment is ongoing, expected to hit 1000 patients by March 2017. The BASE study could potentially aid in focusing the initial diagnostic evaluation to determine stroke etiology, with more rapidly initiated targeted evaluations and secondary prevention strategies.

Clinical Trial Registration

Clinicaltrials.gov NCT02014896 https://clinicaltrials.gov/ct2/show/NCT02014896?term=biomarkers+of+acute+stroke+etiology&rank=1

## Introduction

Acute ischemic stroke (AIS) remains a leading cause of mortality and morbidity in the USA, affecting over 800,000 adults annually and leaving many with permanent disability [1]. Furthermore, hundreds of thousands of Americans experience a transient ischemic attack which often precedes a major stroke and serves as a warning for future ischemic events [2]. Despite resolved symptoms, experiencing a TIA (transient ischemic attack) is associated with a stroke risk of up to 20% within 90 days. Collectively, previous stroke and TIA confer an annual recurrent stroke risk of 3–4% [1]. Emergent evaluation, prompt acute treatment, and identification of stroke or TIA etiology for specific secondary prevention are critical for decreasing further morbidity and mortality of cerebrovascular disease [2].

Key to secondary prevention is stroke etiology identification. This is because the stroke etiology determines which treatment is most effective to prevent future strokes. In addition to risk factor modification for all patients with stroke or TIA, anticoagulant therapy is indicated for cardioembolic stroke. This is in contradistinction to atherogenic strokes where antiplatelet agents are recommended. Currently, the diagnosis of ischemic stroke etiology is determined from a combination of patient history, clinical assessment, cerebrovascular imaging, and cardiovascular evaluation. However, even with extensive testing, identifying the cause of an acute stroke is challenging. Strokes of unclear etiology, or cryptogenic strokes, represent a significant risk as optimal prevention measures cannot be identified. Therefore, there is a great need to identify the pathogenesis of acute ischemic stroke in order to implement targeted and effective preventative measures. Recent studies have suggested whole blood RNA expression may help differentiate ischemic stroke mechanisms [3–7].The Biomarkers of Acute Stroke Etiology (BASE) study (NCT02014896) is a multicenter observational study utilizing RNA gene expression to identify the etiology of acute ischemic stroke. When a stroke or TIA occurs, the immune system changes gene expression in multiple cell types, thus activating innate and adaptive immune responses. Previous studies suggest that differential gene expression profiles are a function of stroke subtype, [3–7] with each subtype producing a unique gene expression “signature”. The Ischemia Care diagnostic platform consists of whole blood biomarker tests to determine the etiology of ischemic stroke (ISCDX, Blue Ash, OH) by measuring acute ischemic stroke gene expression changes. For example, the ISCDX test distinguishes between cardioembolic and large artery, as well as lacunar, atherosclerotic stroke using a signature of 40 unique genes. A patient’s pattern of gene regulation can determine if the stroke etiology is that of a cardioembolic or large artery atherosclerotic source. Further, a separate 37 gene signature can differentiate cardioembolic strokes caused by atrial fibrillation (AF) or other cardioembolic sources. Ultimately, for most patients, the diagnostic expression pattern clearly identifies stroke etiology.

The primary objective of the BASE study is to confirm the diagnostic accuracy of the ISCDX test to identify stroke subtypes in patients with acute ischemic stroke. This manuscript describes the methodology employed in the BASE study to identify stroke etiology in patients presenting with acute stroke.

## Methods

BASE is an ongoing prospective multicenter convenience sample study, registered as NCT02014896 and approved by each participating Institutional Review Board. Patients with acute ischemic stroke who meet the inclusion and exclusion criteria (Table 1) are enrolled in the Emergency Department (ED) and blood samples are drawn. Control samples consist of 100 non-stroke ED patients matched on clinical risk factors of age, race, gender, smoking history, diabetes, hypertension, atrial fibrillation, and hyperlipidemia.

**Table 1:** Inclusion and exclusion criteria

Inclusion criteria
Suspected acute ischemic stroke within 24 (+/− 6) hours of symptom onset
Baseline CT normal, without hemorrhage or alternate explanation for symptoms
>18 years old
Informed consent obtained
Exclusion criteria
Central nervous system infection within 30 days
Serious head trauma within 30 days
Any stroke within 30 days
Active cancer (not in remission)
Autoimmune disease (e.g., lupus)
Acute systemic infection
Major surgery within 90 days

BASE initially enrolled acute stroke patients within 8 h of symptom onset or the time of last known to be normal. However, after patient enrollment reached 650, evaluation time was lengthened to 24 (+/−6) hours. This was from a planned interim data analysis determining the 24 (+/−6) hour window from symptom onset was most predictive for identifying stroke cause using blood biomarkers, was most consistent with the time a stroke patient would present, and represented the window for which a blood test for stroke would be used clinically.

Typically, prior to enrollment, patients are evaluated by the local stroke team or ED physicians, have undergone baseline laboratory testing and cerebrovascular imaging, and may receive intravenous thrombolysis and/or endovascular therapies.

Approximately 2.5 mL of blood is drawn into two PreAnalytiX® PAXgene® blood RNA tubes (Qiagen) within 24 (+/−6) hours of stroke onset. Additional draws occur at 24 (+/− 6), and 48 h (+/− 6), or at ED/hospital discharge, whichever comes first. Longer collection periods were considered but were challenged by the amount of RNA response making it difficult to identify diagnostic patterns consistent with the primary objective of this study. Eligible control subjects are patients presenting without a potentially neurologic complaint and have blood drawn within 6 h of ED presentation.

PAXgene tubes can be kept at room temperature for up to 24 h, and then are frozen at −20 °C, until shipped on dry ice to the Ischemia Care CLIA laboratory (Blue Ash, OH) where the ISCDX testing is performed. The entire sample from one tube will be used to perform the ISCDX test. The second tube is stored at −80 °C for future testing.

Analysis for RNA expression is performed by Affymetrix® human gene ST array plates. These provide whole-genome coverage, including protein coding and long intergenic non-coding RNA (lincRNA) transcripts. Whole genome arrays thus have the ability to provide a complete profile of mRNA expression. The microarray procedure is performed as follows:

1. RNA extraction: Total RNA is extracted from blood collected in PAXgene RNA tubes which are used to specifically preserve the integrity of the RNA.
2. cDNA synthesis and labeling: Multiple complementary DNA (cDNA) copies are made of each RNA. cDNAs are fragmented to sizes for optimal hybridization to the probes on the microarrays and labeled so they can be stained and detected after hybridization to the microarray.
3. Microarray hybridization: Amplified, fragmented and labeled cDNAs are hybridized overnight to an Affymetrix U133 plus 2.0 microarray. Each microarray contains probes for the majority of expressed RNAs from the human genome.
4. Microarray staining and scanning: After hybridization, each microarray is washed, stained with fluorescence that binds to the labels previously attached to each cDNA, then rinsed. Each microarray is then scanned with a laser to record the level of expression of each probe on the array.
5. Microarray data is normalized using the “Signal Space Transformation with probe Guanine Cytosine Count Correction” algorithm (ThermoFisher, Waltham, MA) to control for normal experimental variability. Array quality control metrics are checked against acceptable ranges and rejected when not within range. As an additional quality check, sex-specific expressed genes are checked against the clinically recorded sex to identify potential sample mix-ups.

All study patients undergo standard clinical assessments, with baseline biochemistry and neuroimaging as standard of care for acute stroke. Vascular imaging is not required before enrollment. Control patients do not receive specified imaging or biochemistry assays as part of the study protocol. The diagnostic evaluation is per standard of care and is up to the physician team caring for the patient. In the USA, this generally consists of a 12-lead ECG, bedside ECG monitoring, standard transthoracic echocardiogram (or transesophageal echocardiogram as indicated), and outpatient cardiac event monitoring (the duration of which is determined by the physician of record).

Specific study purpose data include demographics, past medical history, social history, medications, ED evaluation information (including the National Institutes of Health stroke scale), neurologic symptom duration and onset time, baseline cerebrovascular imaging, laboratory tests, electrocardiogram, and cardiac monitoring. Most acute ischemic strokes are admitted per local protocol and receive care per local treating physicians. Cardiac evaluations during admission are collected, as will data of any other therapy or studies performed to treat or determine stroke etiology.

Ischemic stroke etiology is determined locally using all sources of clinical information, according to the Trial of Org 10172 in Acute Stroke Treatment (TOAST) classification (Table 2) [8]. TOAST, (Table 2), is a well validated classification system with 5 subtypes of ischemic stroke. A cardioembolic stroke diagnosis requires at least one source of cardiac emboli and the exclusion of large or small vessel causes of stroke. Cardioembolic sources include AF, acute myocardial infarction, prosthetic valves, and/or cardiomyopathy. Patients with AF are identified using electrocardiogram, echocardiogram, and cardiac monitoring. Cardiac monitoring is performed as standard of care (either during hospitalization or using outpatient event monitoring in unconfirmed suspected AF).

**Table 2:** Subtype of ischemic strokes, TOAST classification

Stroke subtype	Recommended treatment
Large artery atherosclerosis	Antiplatelet therapy
Cardioembolic	Anticoagulation therapy
Small-vessel occlusion (lacune)	Blood pressure control
Stroke of other determined etiology	Therapy should be specific to cause
Stroke of undetermined etiology (cryptogenic)	Aspirin therapy

*TOAST* Trial of Org 10172 in Acute Stroke Treatment

The diagnosis of large-vessel stroke requires >50% stenosis of ipsilateral extracranial or major intracranial artery (middle cerebral artery, posterior cerebral artery, basilar artery) presumed due to atherosclerosis determined by ultrasound, computed tomography angiography, magnetic resonance angiography, or digital subtraction angiography, and is further supported by the absence of acute infarction in other vascular territories. Because of a lack of standardization for defining stroke in the setting of ulcerated plaques of less than <50% narrowing, sites were not asked to categorize these, rather they are placed in the cryptogenic category.

The diagnosis of small-vessel stroke requires symptoms corresponding to a subcortical infarction <15 mm in longest diameter on brain imaging, typically identified on MRI, and the exclusion of other stroke mechanisms. Often, patients will present with classic lacunar syndromes (pure motor hemiparesis). Stroke caused by other uncommon etiologies refers to atypical but specific causes of ischemic stroke (e.g., nonatherosclerotic arteriopathies, vascular dissections, or hypercoagulable states).

Finally, strokes with an extensive work up and remaining of unknown origin are referred to as cryptogenic, patients with multiple stroke etiologies identified are placed in their own category, and those with insufficient information are categorized as such.

The final gold standard diagnosis is determined by an adjudication committee, blinded to ICDX testing results, and consisting of two vascular neurologists independently reviewing all available data. All diagnostic impression data will be used for the TOAST criteria. In cases of unresolved diagnostic disagreement, a third vascular neurologist will serve as a tie breaker.

The BASE study is non-interventional, with treating physicians blinded to genomic test results, thus presenting minimal to no patient risk. Categorical data will be analyzed by chi-square testing and linear continuous data by student’s *t* test. Univariate analysis will identify significant outcome predictors for multivariable modeling. RNA expression data will be presented as heat maps with multiple comparison corrections. Statistical performance of cut points, determined by multivariable modeling and by heat map identification, will be presented using sensitivity, specificity, and *C* statistic, as well as positive and negative predictive values and likelihood ratios. Net reclassification improvement and integrated discrimination improvement will evaluate the change in clinical diagnosis using RNA expression.

The robust BASE trial methodology is predicted to allow for the generation of a number of RNA expression signatures that will be of clinical significance. First will be differentiating between strokes resulting from embolic causes, as compared to stroke caused by large artery thrombosis. This important determination dichotomizes treatment strategies into the divergent categories of either anticoagulants or antiplatelet medication. Secondly, RNA expression signatures are likely to separate true stroke presentations from that of stroke mimic. As a point of care test, this distinction would have massive health care economic benefits as the current standard results in patients suffering from stroke mimics receive expensive, complicated, and unnecessary evaluations that are ultimately negative. A third potential outcome from the BASE trial is that of determining the “last known normal status”. Per current guidelines, the administration of lytic therapy requires a confirmed stroke onset of less than 4.5 h. In all patients, awakening from sleep, the “last known normal” parameter is unknown. This results in 80% of patients with acute stroke not being able to receive life-saving lytic therapy. It is anticipated that the RNA expression signature will identify the time of stroke onset, and if developed as a point of care test, could provide the opportunity for many more patients to receive therapy for their acute stroke presentation.

As is the case with all investigations, the potential for methodologic limitations may exist. Because the medical care in patients enrolled in BASE is determined by the physician caring for the patient, and not by a defined protocol, variations in stroke etiology evaluation may occur (e.g., not all patients may receive echocardiographic bubble studies), and how this may impact outcomes will be unmeasured. Furthermore, the timing of RNA expression measurement was arbitrarily chosen at 24 h as this is a time for which marker analysis is obtainable and is still within a clinically relevant window. Arguments for alternative timing could be effected and may provide impetus for future investigations. Finally, the decision to define a vascular stenosis <50% to be more likely associated with embolic events represents a compromise for consistent diagnosis, but may ultimately not be definitive in its accuracy.

BASE is funded by Ischemic Care, Inc., whose involvement includes providing funding for blood and data collection, assay performance, gold standard diagnostic adjudication, and statistical analysis.

## Summary

The BASE study began January 2014. At the time of writing there are 22 recruiting sites. Enrollment is on target, expected to include 1000 patients by March 2017. Results from the BASE trial will result in the identification of a series of unique stroke biomarkers. It is anticipated that the identification of stroke etiology will be possible, thus differentiating large artery atherogenic events from stroke caused by emboli. Because this determination is not currently possible, it is estimated that the 250,000 annual US stroke patients whose stroke etiology is unknown, may benefit.
